# Effector Protein Cig2 Decreases Host Tolerance of Infection by Directing Constitutive Fusion of Autophagosomes with the *Coxiella*-Containing Vacuole

**DOI:** 10.1128/mBio.01127-16

**Published:** 2016-07-19

**Authors:** Lara J. Kohler, Shawna R. Reed, Shireen A. Sarraf, David D. Arteaga, Hayley J. Newton, Craig R. Roy

**Affiliations:** a Department of Microbial Pathogenesis, Boyer Center for Molecular Medicine, Yale University School of Medicine, New Haven, Connecticut, USA; b Biochemistry Section, Surgical Neurology Branch, National Institute of Neurological Disorders and Stroke, National Institutes of Health, Bethesda, Maryland, USA; c Department of Microbiology and Immunology, University of Melbourne, at the Peter Doherty Institute for Infection and Immunity, Melbourne, Victoria, Australia

## Abstract

*Coxiella burnetii* replicates in an acidified lysosome-derived vacuole. Biogenesis of the *Coxiella*-containing vacuole (CCV) requires bacterial effector proteins delivered into host cells by the Dot/Icm secretion system. Genetic and cell biological analysis revealed that an effector protein called Cig2 promotes constitutive fusion of autophagosomes with the CCV to maintain this compartment in an autolysosomal stage of maturation. This distinguishes the CCV from other pathogen-containing vacuoles that are targeted by the host autophagy pathway, which typically confers host resistance to infection by delivering the pathogen to a toxic lysosomal environment. By maintaining the CCV in an autolysosomal stage of maturation, Cig2 enabled CCV homotypic fusion and enhanced bacterial virulence in the *Galleria mellonella* (wax moth) model of infection by a mechanism that decreases host tolerance. Thus, *C. burnetii* residence in an autolysosomal organelle alters host tolerance of infection, which indicates that Cig2-dependent manipulation of a lysosome-derived vacuole influences the host response to infection.

## IMPORTANCE

*Coxiella burnetii* is an obligate, intracellular bacterial pathogen that replicates inside a unique, lysosome-like compartment called the *Coxiella*-containing vacuole (CCV). Over 130 bacterial effector proteins are delivered into the host cell cytosol by the *C. burnetii* Dot/Icm type IV secretion system. Although the Dot/Icm system is essential for pathogenesis, the functions of most effectors remain unknown. Here we show that the effector protein Cig2 is essential for converting the CCV to an organelle that is similar to the autolysosome. Cig2 function promotes constitutive fusion between the CCV and autophagosomes generated by selective autophagy. Cig2-directed biogenesis of an autolysosomal vacuole is essential for the unique fusogenic properties of the CCV and for virulence in an animal model of disease. This work highlights how bacterial subversion of the host autophagy pathway can influence the cell biological properties of the CCV and influence the host response to infection.

## INTRODUCTION

*Coxiella burnetii* is an obligate, intracellular, bacterial pathogen that is the causative agent of Q fever, which is a flu-like illness that can occur when humans are exposed to *C. burnetii* shed by infected animals (1, 2). Upon uptake by host alveolar macrophages, *C. burnetii* resides in a vacuole that undergoes endocytic maturation and fusion with lysosomes (3). Acidification of the vacuole containing *C. burnetii* results in activation of the Dot/Icm type IV secretion system (T4SS), which delivers a repertoire of over 100 bacterial proteins called “effectors” into the host cell cytosol (4–9). Although the biochemical activities of most effectors remain unknown, the Dot/Icm system is essential for creating a vacuole that supports *C. burnetii* replication (4, 10).

The mature *Coxiella*-containing vacuole (CCV) and lysosomes have shared properties that include an acidic pH, active lysosomal proteases in the lumen, and lysosomal membrane proteins such as LAMP-1 and CD63 (11, 12). CCVs are highly fusogenic with each other and with other organelles of the endocytic pathway. The resulting fusion promotes the formation of a single spacious vacuole that is easily observed by light microscopy (13). In contrast to conventional lysosomes, microtubule-associated protein 1A/1B-light chain 3 (LC3) was found to localize to CCVs (14–16). LC3 is a member of the mammalian Atg8 family of autophagy proteins that are covalently attached to the lipid phosphatidylethanolamine during the process of autophagosome formation (17).

Selective autophagy is a process used to eliminate damaged organelles or protein aggregates from the cytosol. Upon induction of selective autophagy, the targeted cargo is ubiquitinated, and specialized autophagy receptors, such as p62/SQSTM1 or NDP52, then bind both to ubiquitin on the autophagic cargo and to Atg8 proteins on an isolation membrane that eventually envelopes the cargo into an organelle called an autophagosome (18, 19). The conjugation of Atg8 proteins to the preautophagosomal structure (PAS) drives expansion and formation of the isolation membrane (20). The autophagosome generated by selective autophagy is a double-membrane structure that has Atg8 conjugated to both the internal membrane, where Atg8 is exposed to the environment of the lumen, and the external membrane, where Atg8 is exposed to proteins in the cytosol.

The external membrane of the autophagosome fuses with the membrane of a lysosome to create a hybrid organelle called an autolysosome. This fusion event results in the delivery of the internal autophagic vesicle containing cargo called an “autophagic body” into the lumen of the lysosome. Lysosomal enzymes then target the autophagic body for degradation (21, 22). The autolysosome contains lysosomal proteins such as LAMP-1 and autophagosomal proteins such as Atg8. Once the autophagic cargo has been degraded, lysosomes are regenerated (23).

Selective autophagy is important for multiple homeostatic processes in eukaryotic cells; however, the autophagy machinery also contributes to host defense pathways that protect cells against microbial pathogens. Many intracellular pathogens modify the vacuole in which they reside, which often results in these vacuoles being recognized by the host as damaged organelles. This typically results in the ubiquitination of proteins on the membrane of the pathogen-occupied vacuole and recruitment of autophagic receptors, which target the vacuole for a type of selective autophagy referred to as “xenophagy.” Targets of xenophagy are identified by the appearance of autophagic membranes that surround the pathogen, and these membranes are highly enriched for Atg8 proteins such as LC3 (24, 25). There is also a process called “LC3-associated phagocytosis” (LAP), which represents a noncanonical use of the autophagy machinery for host defense (26–28). During LAP, the autophagy machinery directly conjugates LC3 to the cytosolic surface of phagosomes during early endocytic maturation. Unlike a conventional autophagosome, the LC3-containing phagosome generated during LAP has a single membrane, with LC3 restricted to its cytosolic surface (28).

*Coxiella burnetii* transposon insertion mutants deficient in the gene *cig2* (also known as *cvpB* and *C. burnetii* 0021 [*cbu0021*]) reside in vacuoles that do not contain LC3 (29). Additionally, cells infected with these *cig2*-deficient mutants (*cig2::*Tn) display multiple vacuoles per cell. This is in contrast to cells infected with an isogenic wild-type strain of *C. burnetii* in which intracellular bacteria are contained in a single LC3-positive vacuole. Intriguingly, a *cig2* mutant phenotype was also observed when host cells with defects in the autophagy machinery were infected with wild-type *C. burnetii* (29). Because *cig2* encodes an effector protein delivered into host cells by the T4SS (29, 30), these data suggested that Cig2 function is important for promoting an interaction between the host autophagy system and the CCV and that subversion of the autophagy machinery by *C. burnetii* is important for creating the highly fusogenic CCV.

How Cig2 may subvert the host autophagy system remains unknown. The Cig2 protein does not have significant homology to other proteins, and conserved domains are not identifiable in Cig2. Thus, we set out to determine the mechanism by which Cig2 modulates the host autophagy system to generate the mature CCV. We hypothesized that Cig2 function could (i) promote the direct recruitment of the host autophagy machinery to the CCV by a LAP-like process; (ii) generate a signature on the CCV, allowing the host cell to recognize the compartment as a damaged organelle that is targeted for selective autophagy; or (iii) promote the constitutive fusion of conventional autophagosomes with the CCV to maintain this organelle in an autolysosomal stage of maturation.

## RESULTS

### LC3 is abundant in the lumen of the CCV.

**FIG 1:**
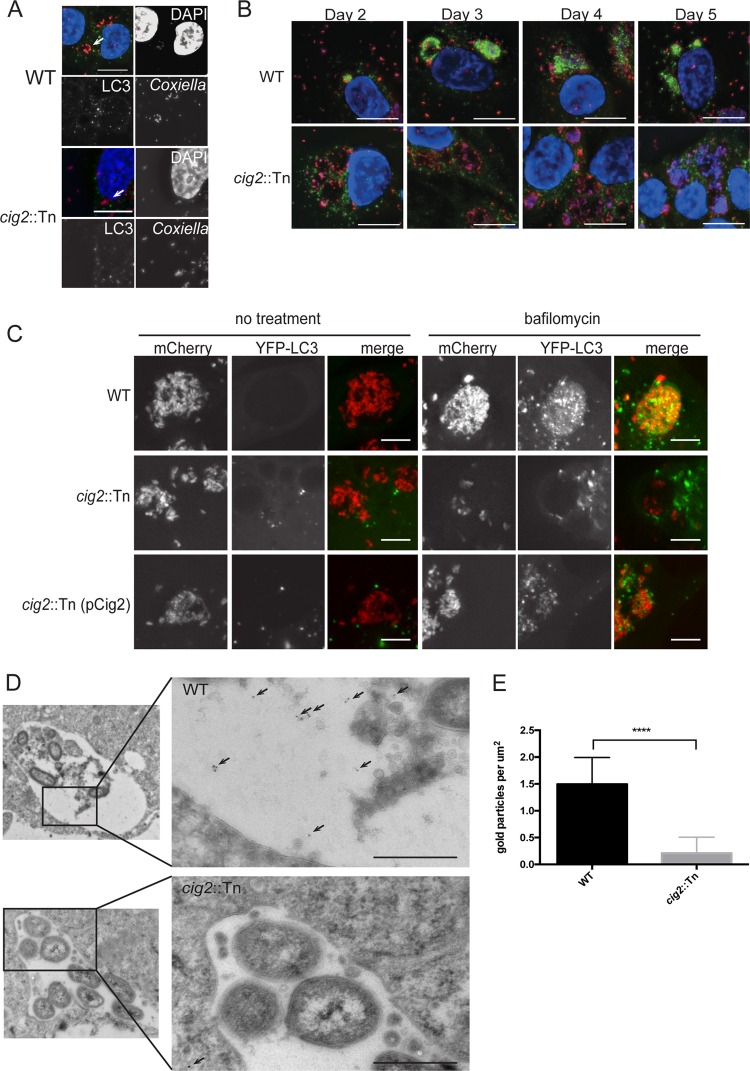
LC3 is abundant in the CCV lumen. (A) Fluorescence micrographs of HeLa cells 24 h after infection with either the parental strain of *C. burnetii* (WT) or the isogenic *cig2*::Tn mutant. Merged panels show staining for LC3 (green), *Coxiella* (red), and DAPI (blue). Bars, 10 µm. (B) Fluorescence micrographs of HeLa cells infected with either the parental strain of *C. burnetii* (WT) or the *cig2*::Tn mutant at the indicated times after infection. Panels show staining for LC3 (green), *Coxiella* (red), and DAPI (blue). Bars, 10 µm. (C) HeLa cells producing YFP-LC3 were infected for 5 days with the indicated strains of *C. burnetii* producing mCherry. Live-cell imaging was used to visualize mCherry (red) and YFP-LC3 (green) in cells that were either left untreated (no treatment) or treated with bafilomycin for 1 h prior to imaging. Bars, 5 µm. (D) Images obtained by cryo-immunoelectron microscopy of HeLa cells producing GFP-RFP-LC3 infected with the indicated *C. burnetii* strains for 5 days. Arrows highlight the locations of anti-GFP-labeled gold particles that colocalized with GFP-RFP-LC3 associated with autophagic bodies in the lumen of vacuoles formed by *C. burnetii* (WT). Bars, 500 nm. (E) The density of gold particles in the lumen of the CCV was determined for 10 different vacuoles and plotted as the average number of gold particles per square micrometer. ****, *P* < 0.0001.

After 5 days of infection, endogenous LC3 localizes to vacuoles containing wild-type *C. burnetii* but does not localize to vacuoles containing *cig2*::Tn mutant bacteria (29). To assess whether LAP-mediated conjugation of LC3 to phagosomes occurs during *C. burnetii* infection, a time course experiment was performed. LC3 was not detected on CCVs formed at 1 day postinfection (Fig. 1A). After 2 days of infection, LC3 was detected on vacuoles formed by wild-type *C. burnetii* but not on vacuoles formed by the *cig2*::Tn mutant (Fig. 1B). LC3 remained associated with vacuoles containing wild-type *C. burnetii* for the duration of infection, whereas LC3 was not detected on vacuoles containing the *cig2*::Tn mutant at any stage of infection. Thus, the recruitment of LC3 to vacuoles containing *C. burnetii* requires the function of Cig2, which is an effector protein that is delivered into host cells only once bacteria have established residence in an acidified late endosomal compartment.

Live-cell imaging further delineated the association and distribution of LC3 on vacuoles containing *C. burnetii*. A stable line of HeLa cells producing a yellow fluorescent protein-LC3 (YFP-LC3) fusion protein was used for the live-cell imaging studies. HeLa YFP-LC3 cells were infected with *C. burnetii* for 5 days. Unexpectedly, YFP-LC3 was not detected on CCVs formed by any of the *C. burnetii* strains tested, even though YFP-LC3 was detected on autophagosomal vesicles in other regions of the infected cells (Fig. 1C). Because YFP fluorescence is quenched inside acidified organelles (31), the inability to detect YFP-LC3 associated with the CCVs could be explained if the endogenous LC3 protein detected by immunofluorescence were to reside primarily in the lumen of the CCV. To test this hypothesis, infected cells were treated with bafilomycin A1 to neutralize the pH inside the CCV and other endosomal compartments. In cells treated with bafilomycin for 60 min, YFP-LC3 was associated with vacuoles containing wild-type *C. burnetii* and with vacuoles containing the complemented mutant *cig2*::Tn (pCig2), but YFP-LC3 was not detected on vacuoles containing the *cig2*::Tn mutant (Fig. 1C). These data suggest that the majority of the LC3 associated with the CCV is located in the lumen of the vacuole and not on the cytosolic surface of the limiting membrane and that LC3 accumulation in the CCV requires Cig2 function.

Cryo-immunoelectron microscopy was used to further resolve the location of LC3 on the CCV in HeLa cells. A HeLa cell line stably expressing green fluorescent protein-red fluorescent protein-LC3 (GFP-RFP-LC3) was infected for 5 days. GFP-RFP-LC3 was detected by immunogold labeling within the lumen of CCVs formed by wild-type *C. burnetii* and was less abundant within CCVs formed by the *cig2*::Tn mutant (Fig. 1D). Measurements of gold particle density confirmed that LC3 was significantly more abundant in the lumen of vacuoles containing wild-type *C. burnetii* than in those containing the *cig2*::Tn mutant (Fig. 1E). Most gold particles were associated with small structures in the lumen of the wild-type CCV, with the structures resembling autophagic bodies delivered into the CCV by fusion of autophagosomes with the vacuole (Fig. 1D). No other differences between vacuoles in the two samples were observed. Thus, three independent methods indicated that Cig2 function was required for robust LC3 association with the CCV. Because LC3 localized to the CCV lumen and was not detected at 1 day after infection, this association suggests the fusion of autophagosomes with the CCV and not direct conjugation of LC3 to the limiting membrane by a process such as LAP.

### Cig2 promotes remodeling of the CCV by selective autophagy.

**FIG 2:**
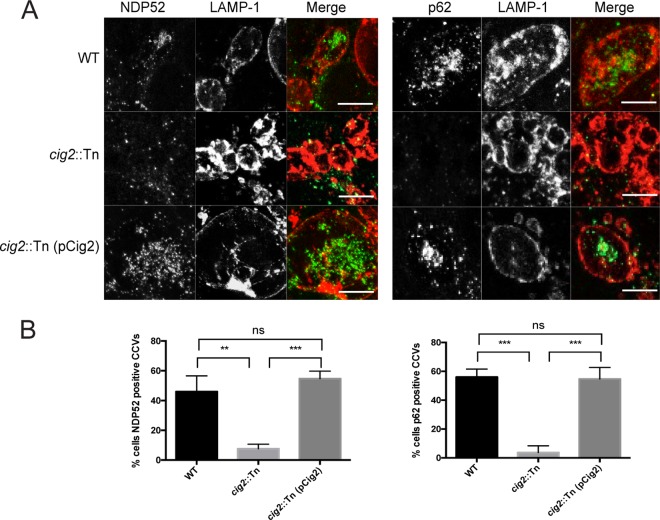
Cig2 is required for the accumulation of autophagy receptors in the CCV. (A) Fluorescence micrographs of HeLa cells 5 days after infection with the parental strain of *C. burnetii* (WT), the *cig2*::Tn mutant, or the complemented cig2::Tn mutant (pCig2). Merged panels show staining for the autophagy receptor NDP52 or p62 (green) and LAMP-1 (red). Bars, 10 µm. (B) Bar graph indicating the percentage of cells containing CCVs that were positive for either NDP52 or p62 among the cells described for panel A. Means of results of three biological replicates ± standard deviations (SD) are shown. ***, *P* < 0.001; **, *P* < 0.01; ns, not significant [unpaired *t* test]). Data are representative of results of three independent experiments.

There are multiple receptor proteins that recognize cargo and target these substrates for receptor-mediated selective autophagy. Because LC3 localized within the CCV lumen was associated with structures predicted to be autophagic bodies, the hypothesis that delivery of LC3 was mediated by the fusion of autophagosomes generated by selective autophagy with the CCV was tested. In addition to the CCV being enriched for LC3, this hypothesis predicts that the lumen of the vacuole should also be enriched for the receptor proteins used to generate the autophagosomes that fused with the CCV. Thus, localization of two different autophagy receptors, NDP52 and p62 (p62/SQSTM1), was determined in cells infected with *C. burnetii*. Fluorescence micrographs showed punctate structures that stained positive for NDP52 or p62 in the cytoplasm of infected cells, which represent autophagosomes formed by selective autophagy (Fig. 2A). NDP52 and p62 staining was evident in the lumen of vacuoles containing wild-type *C. burnetii* and in vacuoles containing the complemented *cig2*::Tn (pCig2) strain (Fig. 2). In contrast, robust NDP52 or p62 staining was not detected in vacuoles containing the *cig2*::Tn mutant. Peripheral localization of p62 and NDP52 was not detected on the membrane of the CCVs, which suggests that the vacuole containing *C. burnetii* is not a direct target for selective autophagy. All of the strains tested, including the *cig2*::Tn mutant, were contained in vacuoles that had lysosomal proteins such as LAMP-1, cathepsin-D, and CD63 (29) (see Fig. S1 in the supplemental material), which indicates that Cig2 is not required for delivery of *C. burnetii* to lysosomes but is required for delivery of autophagic receptors to the CCV. The clear correlation between the localization of LC3 in the CCV and the appearance of proteins used for receptor-mediated autophagy supports the hypothesis that delivery of LC3 to the CCV is the result of fusion of mature autophagosomes generated by selective autophagy with the CCV.

**FIG 3:**
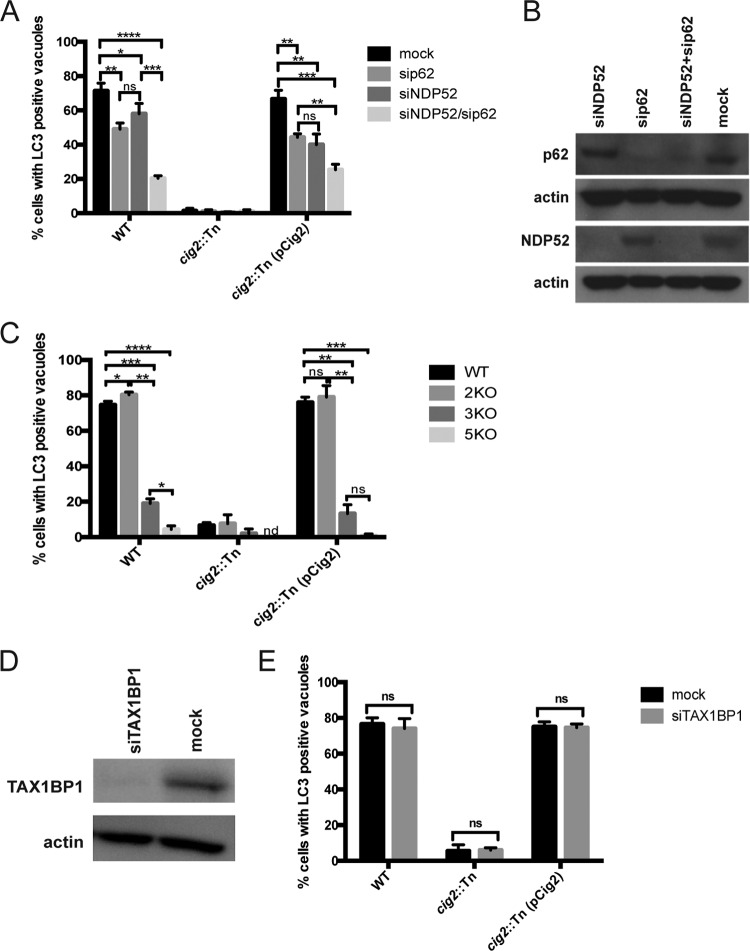
Autophagy receptors accumulate in the CCV. (A) The shading of the bars indicates the autophagy receptors that were silenced before the HeLa cells were infected for 3 days with the strains of *C. burnetii* indicated on the *x* axis. The percentage of infected cells with vacuoles that stained positive for LC3 was determined by fluorescence microscopy. (B) Immunoblot analysis of HeLa cell lysates generated 5 days after transfection of the indicated siRNAs (top labels) shows the levels of depletion of the indicated proteins (left labels). (C) The shading of the bars indicates the HeLa cell line in which genes encoding autophagy receptors were mutated by CRISPR/Cas9 targeting (WT, parental HeLa cells; 2KO, cell deficient in OPTN and NDP52; 3KO, cells deficient in OPTN, NDP52, and TAX1BP1; 5KO, cells deficient in OPTN, NDP52, TAX1BP1, NBR1, and p62). HeLa cells were infected for 5 days with the strains of *C. burnetii* indicated on the *x* axis. The percentage of infected cells with vacuoles that stained positive for LC3 was determined by fluorescence microscopy using an anti-LC3 antibody. The bar graphs in panels A and C depict the means of the results of three biological replicates within the same experiment ± SD. (D) Immunoblot analysis of HeLa cell lysates generated 5 days after transfection with the indicated siRNAs shows depletion of TAX1BP1. (E) Comparison of LC3-positive vacuoles as determined by immunofluorescence microscopy using antibody against endogenous LC3. TAX1BP1 was silenced with siRNA for 2 days, and then cells were infected with the indicated *C. burnetii* strains for 3 days before fixation. Data are representative of the results of two independent experiments. The unpaired *t* test was used to calculate significant differences between samples. ****, *P* < 0.0001; ***, *P* < 0.001; **, *P* < 0.01; *, *P* < 0.05).

If vacuoles containing *C. burnetii* are remodeled by fusion of autophagosomes generated by the selective autophagy system, then interfering with selective autophagy pathways should prevent the delivery of LC3 to the CCV. Autophagy receptor genes were depleted in cells to test this hypothesis. When p62 was silenced, there was a reduction in the number of CCVs that had detectable LC3 (Fig. 3A). A similar reduction in the number of LC3-positive CCVs was observed in cells when NDP52 was silenced. Silencing both NDP52 and p62 resulted in an even greater decrease in the number of LC3-positive CCVs. Immunoblot analysis confirmed that small interfering RNA (siRNA) silencing reduced the expression of the autophagy receptors (Fig. 3B). Thus, a reduction in selective autophagy mediated by the p62 and NDP52 receptors interferes with the delivery of LC3 to vacuoles containing *C. burnetii*, which further indicates that LC3 delivery is mediated by fusion of mature autophagosomes with the CCV.

To independently address whether selective autophagy is important for CCV remodeling by the host autophagy system, a panel of isogenic HeLa cells were used in which genes encoding specific autophagy receptors were sequentially inactivated by clustered regularly interspaced short palindromic repeat (CRISPR)/Cas9-directed mutagenesis (32). There was no detectable defect in LC3 recruitment to vacuoles containing *C. burnetii* in a HeLa cell line in which the genes encoding optineurin (OPTN) and NDP52 were both inactivated (Fig. 3C, 2KO). However, recruitment of LC3 to the CCV was significantly reduced when the gene encoding the TAX1BP autophagy receptor was inactivated next to generate cells deficient in OPTN, NDP52, and TAX1BP1 (Fig. 3C, 3KO). When the genes encoding NBR1 and p62 were inactivated next in addition to those encoding TAX1BP, OPTN, and NDP52, the recruitment of LC3 to the CCV was reduced to the background levels observed for vacuoles containing the *cig2*::Tn mutant (Fig. 3C, 5KO). There was a significant decrease in the number of LC3-positive vacuoles when TAX1BP1 was deleted from cells in which OPTN and NDP52 had previously been deleted. Silencing of TAX1BP1 alone, however, was not sufficient to reduce the number of cells with LC3-positive vacuoles (Fig. 3D and E), which suggests that TAX1BP1 is not essential for formation of autophagosomes that fuse with the CCV. There was a positive relationship between the ability of cells to generate autophagosomes by selective autophagy and recruitment of LC3 to the CCV. These data indicate that selective autophagy is required to generate autophagosomes, which then participate in remodeling the CCV by a process that requires Cig2 function.

### Biogenesis of an autolysosomal vacuole is important for CCV homotypic fusion.

When multiple *C. burnetii* bacteria are internalized by a single host cell, the vacuoles in which they reside undergo homotypic fusion to form a single CCV (13). It was previously shown that multiple CCVs are typically observed in host cells infected with a *cig2*::Tn mutant or in wild-type *C. burnetii*-infected cells in which the autophagy system had been inactivated (29). It remained unclear whether this autophagy-dependent multivacuole phenotype is the result of a defect in homotypic fusion or of enhanced fission of the CCV to generate multiple vacuoles. To address this issue, cells were infected with differentially labeled *cig2*::Tn mutant bacteria. Because homotypic fusion would promote the formation of vacuoles having a mixed population of bacteria, if Cig2 function were important for homotypic fusion, there should be an increase in the number of vacuoles containing a single population of bacteria.

**FIG 4:**
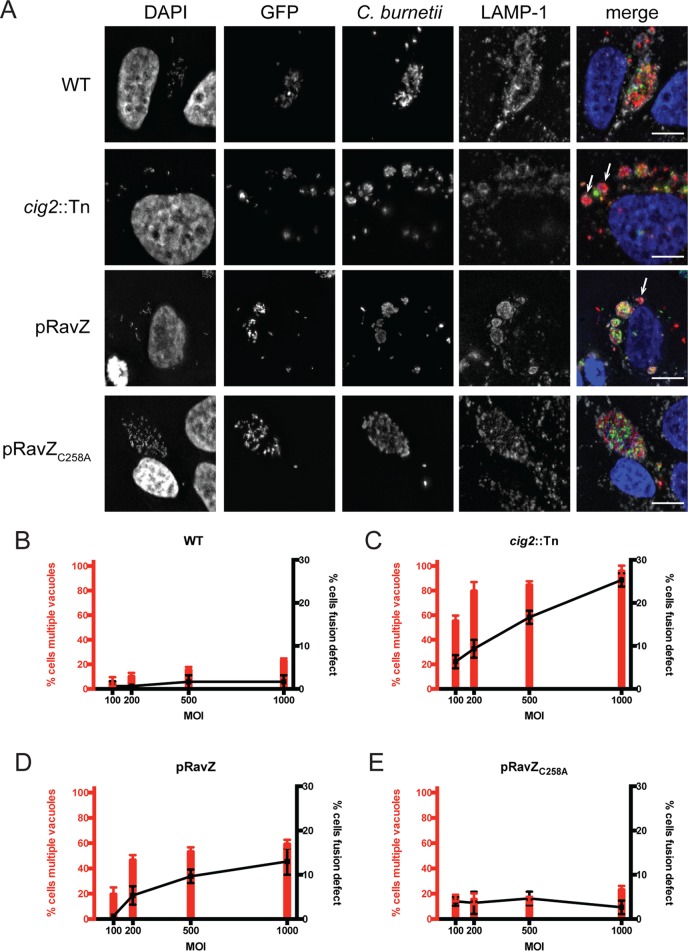
Homotypic fusion of CCVs requires both Cig2 function and host autophagy. (A) Fluorescence micrographs of HeLa cells coinfected with a mixture of equal amounts (MOI of 500) of GFP-positive and GFP-negative variants of the *C. burnetii* strains indicated on the left. Cells were fixed after 5 days of infection. Merged images show staining for DAPI (blue), GFP (green), anti-*Coxiella* antibody (red), and anti-LAMP-1 antibody (gray). Bars, 5 µm. (B to E) HeLa cells were coinfected with a mixture of equal amounts of GFP-positive and GFP-negative variants of the *C. burnetii* strains indicated on top of each graph at the MOIs indicated on the *x* axis of each graph. Cells were fixed after 5 days of infection, and fluorescence microscopy was used to determine the percentage of infected cells that contained multiple vacuoles (left *y* axis and red bars) and the percentage of infected cells that had a fusion defect as defined by the presence of coinfected cells that did not display CCV content mixing (right *y* axis and black squares with connecting lines). Results depict means of the results of three biological replicates ± SD within the same experiment. Data are representative of the results of three independent experiments.

HeLa cells were coinfected with pairs of isogenic *C. burnetii* strains, with the only difference being that one strain contained a plasmid that produced enhanced GFP (EGFP). Control studies demonstrated that EGFP fluorescence is maintained at 5 days postinfection in host cells (see Fig. S2 in the supplemental material). Consistent with previous data, most cells coinfected with the wild-type strain of *C. burnetii* (with or without EGFP) contained a single vacuole, and this was independent of the multiplicity of infection (MOI). The percentages of cells having vacuoles containing both EGFP-positive and EGFP-negative bacteria remained similar as the MOI was increased, which is consistent with homotypic fusion promoting vacuole mixing in cells that had internalized multiple bacteria (Fig. 4A and B). In contrast, there was a positive correlation between the number of cells having multiple vacuoles and the MOI during infection with the *cig2*::Tn mutant (with or without EGFP). Importantly, as the MOI increased, the number of *cig2*::Tn mutant-infected cells with vacuoles that contained only EGFP-positive or EGFP-negative bacteria also increased (Fig. 4A and C). This defect in bacterial content mixing indicates that the multivacuole phenotype displayed by cells infected with the *cig2*::Tn mutant is the result of a homotypic fusion defect. If the multivacuole phenotype were the result of enhanced fission of CCVs but with the fusion still intact, then vacuoles with mixed bacterial content would result.

Previously, it was shown that *C. burnetii* producing the *Legionella pneumophila* effector protein RavZ, which is an Atg8 protease that globally disrupts host autophagy, displays a multivacuole phenotype similar to that of the *cig2*::Tn mutant (29). RavZ is delivered by the *C. burnetii* Dot/Icm system after vacuoles have fused with late endocytic organelles and have been acidified. Thus, *C. burnetii* producing RavZ was used to determine if the acute disruption of autophagy that occurs after *C. burnetii* gains access to a lysosome-derived vacuole would prevent homotypic fusion of the CCV. Isogenic strains of *C. burnetii* producing either RavZ or the catalytically inactive RavZ_C258A_ mutant protein were able to replicate in HeLa cells as efficiently as the wild-type *C. burnetii* strain (see Fig. S3 in the supplemental material). Similarly to the results seen with *cig2*::Tn mutant, as the MOI increased, the number of cells coinfected with *C. burnetii* producing RavZ (with or without EGFP) that contained only EGFP-positive or EGFP-negative bacteria increased and the percentage of cells that had multiple CCVs also increased (Fig. 4A and D). This was dependent on RavZ-mediated inhibition of autophagy, as no differences were observed when *C. burnetii* producing the inactive RavZ_C258A_ protein (with or without EGFP) was compared to wild-type *C. burnetii* (with or without EGFP) (Fig. 4A and E). Because RavZ and Cig2 are both translocated into host cells after the vacuoles containing *C. burnetii* have matured, these data indicate that homotypic fusion requires constitutive biogenesis and fusion of autophagosomes with the CCV and that Cig2 promotes the process of CCV remodeling by directing fusion of autophagosomes with the CCV.

**FIG 5:**
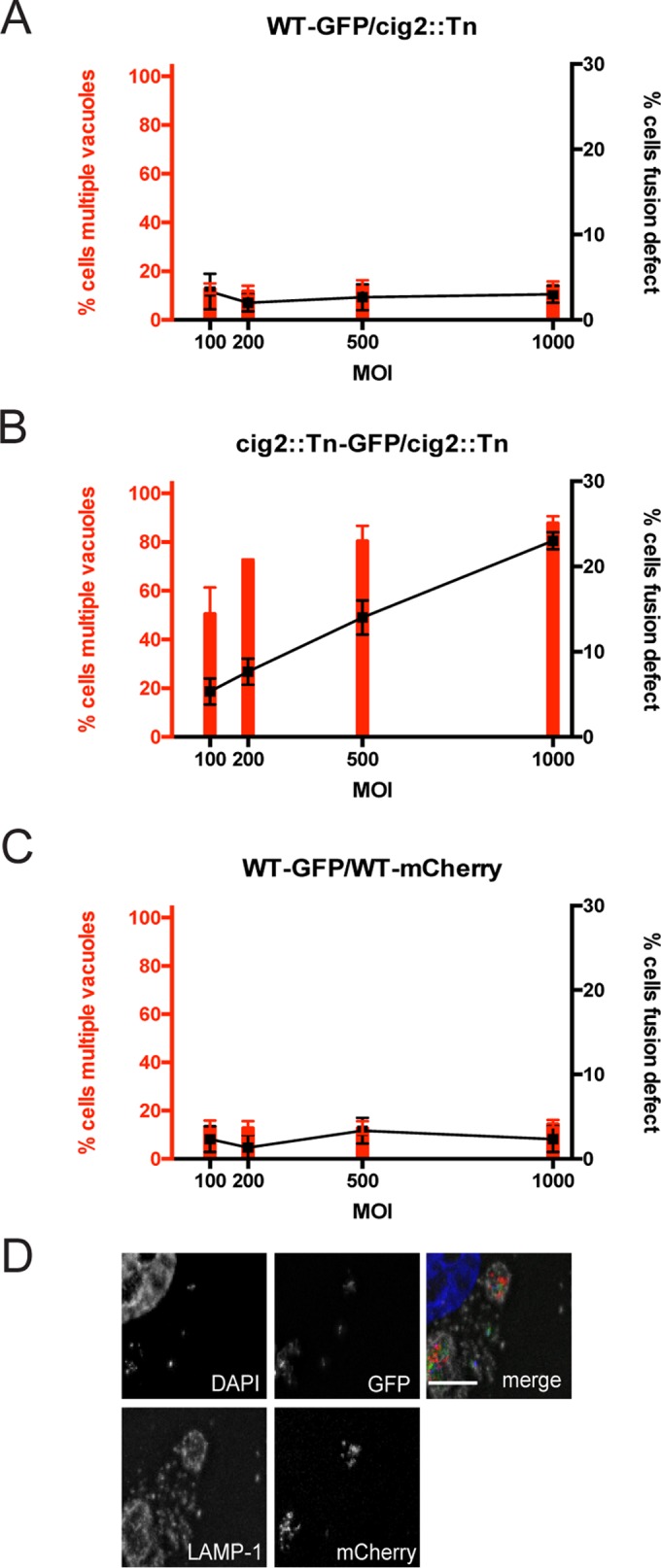
Vacuoles containing wild-type *C. burnetii* fuse with vacuoles containing a *cig2*::Tn mutant. (A to C) HeLa cells were coinfected with a mixture of equal amounts of the fluorescent or nonfluorescent *C. burnetii* strains indicated on top of each graph at the MOIs indicated on the *x* axis of each graph. Cells were fixed after 5 days of infection, and fluorescence microscopy was used to determine the percentage of infected cells that contained multiple vacuoles (left *y* axis and red bars) and the percentage of infected cells that had a fusion defect as defined by coinfected cells that did not display CCV content mixing (right *y* axis and black squares with connecting lines). Results depict means of the results of three biological replicates ± SD within the same experiment. Data are representative of the results of three independent experiments. (D) Representative fluorescence micrograph of HeLa cells coinfected with a mixture of equal amounts (MOI of 500) of wild-type *C. burnetii* expressing GFP and the GFP-negative *cig2*::Tn mutant. Cells were fixed after 5 days of infection. Merged images show staining for DAPI (blue), GFP (green), anti-*Coxiella* antibody (red), and anti-LAMP-1 antibody (gray). Bar, 5 µm.

Host cells were also coinfected with *C. burnetii* producing EGFP and a *cig2*::Tn mutant producing mCherry to test whether vacuoles containing the *cig2*::Tn mutant could fuse with vacuoles containing wild-type *C. burnetii*. The multivacuole phenotype displayed by the *cig2*::Tn mutant was suppressed by coinfection with wild-type *C. burnetii*. The majority of cells infected with both the *cig2*::Tn mutant and wild-type *C. burnetii* contained single vacuoles with mixed bacterial content even as the MOI was increased, in similarity to the results seen when cells were coinfected with wild-type *C. burnetii* producing EGFP and an isogenic strain producing mCherry (Fig. 5). Thus, autophagy subversion by Cig2 generates an autolysosomal CCV that is highly fusogenic with other lysosome-derived organelles in the cell, which includes vacuoles containing a *cig2*::Tn mutant.

### Cig2 contributes to *C. burnetii* virulence.

The *cig2*::Tn mutant has a distinct vacuole morphology defect but does not have a detectable intracellular replication defect in host cells cultured *ex vivo* (29). All *C. burnetii* genomes sequenced to date have a *cig2* gene predicted to encode a functional protein, whereas many other T4SS effectors are truncated or even annotated as pseudogenes in some of the sequenced *C. burnetii* isolates (4, 29). This suggests that Cig2-mediated subversion of the host autophagy system is important for an aspect of *C. burnetii* infection that cannot be assessed simply by measuring replication *ex vivo*. The *C. burnetii* Nine Mile phase II (NMII) strain was recently shown to productively infect and kill *Galleria mellonella* (wax moth) larvae by a mechanism that required Dot/Icm function (33, 34). Thus, the *Galleria* model was used to determine if *cig2* contributes to *in vivo* infection.

**FIG 6:**
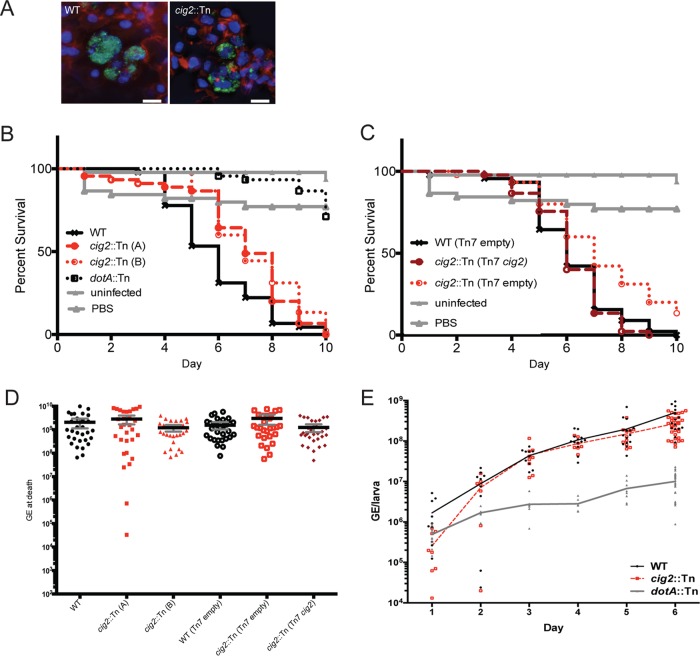
Cig2 function decreases host tolerance of *C. burnetii* infection. (A) Images of hemocytes isolated from *G. mellonella* larvae that were infected with either the parental strain of *C. burnetii* producing GFP or an isogenic *cig2*::Tn mutant strain. Images show *C. burnetii* (green), actin (red), and DNA (DAPI). Bars, 10 µm. (B and C) Curves indicate the percentage of surviving *G. mellonella* larvae at each day after infection with the indicated strains of *C. burnetii*. “*cig2::*Tn (A)” and “*cig2::*Tn (B)” denote two independent *cig2*::Tn mutants. Data represent the averages of the results of three independent experiments in which an individual strain of *C. burnetii* was used to infect 15 larvae. (D) *C. burnetii* numbers were determined by measuring genome equivalents in samples from infected *G. mellonella* larvae at the time of death. Each separate symbol represents data from an infected individual. Data were averaged from results of two independent experiments. (E) *C. burnetii* numbers were determined by measuring genome equivalents in larvae each day after infection. Each symbol represents *C. burnetii* numbers obtained from an individual larva.

Hemocytes recovered from larvae infected with either wild-type *C. burnetii* or the isogenic *cig2*::Tn mutant were analyzed by microscopy. Representative images show that wild-type *C. burnetii* localized to large single vacuoles inside the isolated hemocytes and that, in animals infected with the *cig2*::Tn mutant, the isolated hemocytes contained multiple CCVs (Fig. 6A). Thus, in similarity to what occurs in mammalian cells cultured *ex vivo*, the *cig2* gene is essential for homotypic fusion of CCVs following uptake by *G. mellonella* hemocytes during the course of animal infection.

To assess virulence, a lethal dose (10^6^ genome equivalents per larva) of *C. burnetii* was administered and larval survival was monitored for 10 days. The mean time to death was significantly longer for larvae infected with *C. burnetii* deficient in Cig2 function than for those infected with the wild type (Fig. 6B and Table 1). This reduction in virulence was validated for two independent *cig2*::Tn mutants with insertions in different regions of the *cig2* gene. Thus, Cig2 function is important for virulence in the *G. mellonella* model of infection.

**TABLE 1:** *G. mellonella* infection statistics^a^

Comparison	Survival curve log rank (Mantel-Cox) *P* value	Survival curve *t* test *P* value	Mean GE at death *t* test *P* value	GE at death on day 6 *t* test *P*value
WT versus *cig2*::Tn (A)	0.0042 (**)	0.014 (*)	0.2864 (ns)	0.0952 (ns)
WT versus *cig2*::Tn (B)	0.0005 (***)	0.0001 (***)	0.0867 (ns)	0.6305 (ns)
*cig2*::Tn (A) versus *cig2*::Tn (B)	0.433 (ns)	0.3243 (ns)	0.0086 (**)	0.0405 (*)
WT (Tn*7* empty) versus *cig2*::Tn (Tn*7* empty)	0.0019 (**)	0.0084 (**)	0.0499 (*)	0.5446 (ns)
WT (Tn*7* empty) versus *cig2*::Tn (Tn*7*::*cig2*)	0.656 (ns)	0.9431 (ns)	0.2947 (ns)	0.7875 (ns)
*cig2*::Tn (Tn*7* empty) versus *cig2*::Tn (Tn*7*::*cig2*)	0.0006 (***)	0.0039 (**)	0.0162 (*)	0.3932 (ns)

a Statistics on survival curve and genome equivalent data are presented in Fig. 6B to D. ns, not significant.

For complementation studies, a single copy of the wild-type *cig2* gene under the control of the endogenous promoter was introduced at a heterologous chromosomal location in *trans* to the *cig2*::Tn mutation (annotated as *cig2*::Tn [Tn*7*::*cig2*]) using a Tn*7*-based plasmid integration system (35). Isogenic strains of wild-type *C. burnetii* and the *cig2*::Tn mutant containing only a kanamycin (Kan) resistance cassette integrated at the attnTn*7* site (annotated as the wild type [WT] [Tn*7* empty] and *cig2*::Tn [Tn*7* empty], respectively) were used as control strains. Similarly to what was observed using the parental *cig2*::Tn mutant, larvae infected with the *cig2*::Tn (Tn*7* empty) strain had a significantly longer time to death than the larvae infected with the control WT strain (Tn*7* empty) (Fig. 6C and Table 1). No significant difference was observed with the control WT strain (Tn*7* empty) compared to the *cig2*::Tn (Tn*7*::*cig2*) complemented mutant strain (Fig. 6C and Table 1). Thus, the virulence defect displayed by the *cig2::*Tn mutant was complemented by introduction of a functional *cig2* gene in *trans*.

To determine if Cig2 function affects bacterial replication *in vivo*, the genome equivalents (GEs) for *C. burnetii* were determined for larvae collected at the time of death in two independent experiments (Fig. 6D). This analysis demonstrated robust replication of *C. burnetii in vivo*, but the total numbers of GEs in the larvae killed by wild-type *C. burnetii* and complemented *cig2*::Tn strains were similar to the total numbers of GEs in the larvae killed by the *cig2*::Tn mutants (Fig. 6D and Table 1). Similarly, there was no significant difference in GEs from larvae infected with WT or *cig2*::Tn for larvae collected randomly, not just as dead individuals, over the course of infection (Fig. 6E). Thus, Cig2 function enhances *C. burnetii* virulence in this model, but, similarly to the *ex vivo* results, Cig2 function is not required for intracellular replication.

## DISCUSSION

It was shown previously that the presence of multiple CCVs in an individual host cell was a phenotype observed when autophagy-deficient host cells were infected with wild-type *C. burnetii* and when wild-type host cells were infected with a *cig2* mutant (29). It was also demonstrated that endogenous LC3 localized to the CCV by a mechanism that requires Cig2 function but that the levels of LC3 lipidation in *C. burnetii*-infected cells are not affected by Cig2 function (29). Thus, the Cig2-dependent mechanism by which LC3 is recruited to the CCV remained unknown. Here, this issue was addressed by examining possible membrane transport pathways that could result in LC3 accumulation on a pathogen-occupied vacuole.

Given the importance of LAP in promoting lysosomal maturation and acidification of phagosomes containing microbial pathogens, the possibility that Cig2 may subvert the LAP pathway was attractive because rapid acidification of the CCV would be beneficial to productive infection by *C. burnetii*. One of the hallmarks of LAP is the rapid conjugation of LC3 to the cytosolic surface of a phagosome membrane (28). Time course studies were conducted to determine when LC3 association was detected on the CCV, and there was no evidence of LC3 localization to the CCV before lysosomal maturation had occurred. Additionally, the majority of LC3 associated with the CCV was present in the lumen of the vacuole and not on the cytosolic surface of the limiting membrane. These data indicate that Cig2-dependent recruitment of LC3 to the CCV is unlikely to be due to subversion of the LAP pathway. Because LAP is activated when innate immune receptors such as Toll-like receptor 2 (TLR2) are activated during macrophage phagocytosis (28), and because TLR2 is one of the few innate immune receptors stimulated during *C. burnetii* infection (36), it remains possible that LAP could enhance CCV biogenesis during infection of macrophages. However, it is unlikely that *C. burnetii* effectors would be involved in LAP activation given that the Dot/Icm system does not initiate effector translocation until the CCV has been acidified (37).

For a number of pathogen-occupied vacuoles, the localization of LC3 is an indication that the vacuole is being targeted by the selective autophagy system by a process called xenophagy. One of the hallmarks of xenophagy is the expansion of LC3-positive autophagosomal membranes around the pathogen-occupied vacuole, which results in the envelopment of the vacuole in an autophagic vesicle. Similarly to phagosomes generated by LAP, membranes formed during xenophagy have abundant amounts of LC3 conjugated to the cytosolic surface. Additionally, a vesicle formed by xenophagy has the classic double-membrane structure displayed by conventional autophagosomes (38). These primary characteristics of vacuoles targeted by xenophagy were not apparent on vacuoles containing *C. burnetii*. LC3 localization to the cytosolic surface of the CCV was undetectable in host cells producing YFP-LC3, which is inconsistent with the CCV being a target of host xenophagy. Additionally, electron microscopy (EM) analysis in this study and in previous studies (39, 40) has not identified double-membrane structures enveloping the CCV, which suggests that the vacuole is not being targeted for selective autophagy. Lastly, autophagy receptors have been detected on the limiting membrane of vacuoles being targeted for xenophagy (41) but were not detected on the limiting membrane of the CCV. Thus, unlike other pathogen-occupied vacuoles that have autophagic properties, the CCV is unique in that this compartment is not being recognized as cargo being targeted for envelopment by the selective autophagy pathway.

The data here indicate that LC3 acquisition by the CCV is the result of fusion of mature autophagosomes with the lysosome-derived vacuole in which *C. burnetii* resides. This conclusion is supported by data showing that the CCV lumen accumulates LC3-positive autophagic bodies that have been delivered to the lysosome-derived vacuole by fusion of mature autophagosomes with the CCV. This model is supported by previous EM studies that showed fusion of double-membrane-bound structures with the CCV (42). Previous studies in which fluorescently tagged LC3 was overproduced in mammalian cells have also shown the appearance of LC3 on the limiting membrane of the CCV (14–16, 42). Although we did not detect LC3 on the limiting membrane of the CCV, the fusion of autophagosomes with a lysosome-derived organelle would deliver LC3 to the limiting membrane of the autolysosomal organelle, where LC3 would then be removed by cellular proteases such as ATG4 in the cytosol. Thus, the ability to detect LC3 on the limiting membrane of the CCV likely requires a high level of LC3 expression and low rates of LC3 turnover by ATG4. The lumen of the CCV was also enriched for autophagic receptors that mediate the formation of autophagosomes generated by selective autophagy. Indeed, LC3 localization to the CCV was dependent on autophagosomes generated by selective autophagy because sequential elimination of autophagic receptors that generate these autophagosomes resulted in a proportional decrease in the acquisition of LC3 by the CCV. Thus, the CCV represents an acceptor compartment that fuses with mature autophagosomes that have already captured cargo targeted by the selective autophagy system.

Importantly, the Cig2 effector protein was necessary for continued fusion of autophagosomes with the lysosome-derived vacuole in which *C. burnetii* resides. Cig2 may mimic a host membrane transport protein that promotes fusion of autophagosomes with lysosomes. Alternatively, because fusion of autophagosomes with lysosome-derived vesicles represents the canonical route by which cargo targeted by selective autophagy is delivered to lysosomes for degradation, Cig2 could potentially be disrupting a homeostatic process that terminates fusion of autophagosomes with an autolysosomal compartment. Controlling the extent of fusion of autophagosomes with lysosomes is a process that would enable the organelle to efficiently degrade and recycle nutrients that it has acquired. A recent study suggested that overexpression of Cig2 alters phosphoinositide metabolism on endosomal organelles, perhaps by interfering with a host kinase that generates PI5P on late endosomes (43), but how Cig2 may mediate this effect remains unknown. Because there is very little known about how the size and capacity of an autolysosome are regulated, understanding the biochemical function of Cig2 may provide insight into the dynamics of autophagosome fusion with lysosomes.

One of the consequences of Cig2 function is that the CCV is maintained in an autolysosomal stage of maturation, which is necessary for this organelle to undergo homotypic fusion and to create a single CCV in cells that have internalized multiple bacteria. It has been shown previously that autophagy induces the consumption of lysosomes to generate autolysosomal organelles and that autolysosomal organelles are inherently highly fusogenic (44). Thus, the dependency on Cig2 to promote homotypic fusion of CCVs may be a secondary effect of locking the CCV in an autolysosomal stage of maturation, which means that CCV homotypic fusion is a consequence of the normal fusogenic properties displayed by autolysosomes. This would explain why CCV homotypic fusion requires both Cig2 function and host genes that mediate the biogenesis and fusion of autophagosomes with lysosomes.

Similarly to *Legionella pneumophila* effectors, there is extensive plasticity in the effector repertoire encoded by individual strains of *C. burnetii* (4). Data from genomic sequences of *C. burnetii* strains obtained from humans with different clinical presentations and from infected animals, however, indicate that the *cig2* gene is highly conserved. This suggests that Cig2 function has an important role in the life cycle of *C. burnetii*. However, replication of *C. burnetii* in host cells cultured *ex vivo* does not appear to require Cig2 function even though *cig2* mutants display a clear defect in vacuole biogenesis.

Tolerance has emerged as an important determinant in host survival of microbial infection. Tolerance is defined as the ability of the host to endure the pathological consequences associated with microbial infection. Intriguingly, how infection impacts host metabolism has been shown to dramatically influence tolerance. For instance, infection of fruit flies with *Salmonella enterica* serovar Typhimurium induces an anorexic response that enhances tolerance of infection (45). *Ex vivo* data showing that Cig2 is not essential for intracellular replication of *C. burnetii* suggested that autophagy subversion by Cig2 could potentially impact a host response to infection that influences tolerance. Thus, *C. burnetii* infection was analyzed using the *G. mellonella* model to test whether Cig2 affected virulence. These data revealed a virulence defect for two independent *cig2*::Tn mutants that was complemented by returning wild-type *cig2* to a different chromosomal location. This defect in virulence was not due to a significant difference between the bacterial load of *cig2*::Tn mutants and the parental *C. burnetii* load in infected animals, which indicates that the host was more tolerant of infection by the *cig2*::Tn mutant than of that by the parental strain of *C. burnetii*.

How Cig2 affects host tolerance remains unknown. Given that Cig2 function promotes formation of a vacuole that is locked in an autolysosomal stage of maturation, it is likely that there are differences between the metabolic programs of cells infected with the *cig2*::Tn mutant and those of cells infected with the parental strain of *C. burnetii*. This could influence survival by altering the host cell response to infection. Metabolite concentrations inside cells can influence a variety of innate immune signaling pathways that govern tolerance. For instance, it has been shown that 5′-methylthioadenosine levels dramatically influence host cell pyroptosis in response to infection by *S*. Typhimurium (46). Interfering with bacterial dissemination by the process of melanization can also be affected by changes in host metabolism and can influence tolerance (47). Thus, it is exciting to find that Cig2 can influence host tolerance of infection by maintaining the vacuole in an autolysosomal stage of maturation. Identifying the pathways that contribute to host tolerance that are altered by a Cig2-dependent mechanism during *C. burnetii* infection should provide an important conceptual advance in understanding why autophagic regulation is intimately linked to host survival during microbial infection.

## MATERIALS AND METHODS

### Bacterial strains and host cell lines.

Wild-type Nine Mile phase II (NMII) *C. burnetii* (RSA493) was plaque purified in HeLa cells and then grown axenically in ACCM-2 media as previously described (48, 49). The generation of *C. burnetii* strains expressing *cig2*::Tn and RavZ was previously described (29). Genetically modified *C. burnetii* strains were grown in ACCM-2 with 3 µg/ml chloramphenicol and/or 375 µg/ml kanamycin when appropriate. *C. burnetii* strains were grown for 6 days and then centrifuged for 15 min at 4,000 rpm in a tabletop centrifuge (Eppendorf). Pellets were then resuspended in 5% fetal bovine serum (FBS)–Dulbecco’s modified Eagle’s medium (DMEM) (Gibco). Bacterial genome equivalents were determined by quantitative PCR (qPCR) as previously described (29). HeLa cells were cultured in 10% heat-inactivated FBS (Sigma)–DMEM (Gibco) at 37°C with 5% CO_2_. Stable YFP-LC3 and GFP-RFP-LC3 HeLa cells were generously provided by the laboratory of Tom Melia (Yale). YFP-LC3 HeLa cells were cultured in 10% FBS–DMEM with 100 µg/ml hygromycin B, 200 µg/ml geneticin, and 100 ng/ml doxycycline to maintain the YFP-LC3 plasmid. GFP-RFP-LC3 HeLa cells were grown in 200 µg/ml geneticin–10% FBS–DMEM.

### Indirect immunofluorescence of LC3, receptor proteins, and CD63.

HeLa cells were plated in 5% FBS–DMEM at 2 × 10^4^ cells per well on untreated coverslips in a 24-well dish on the day of infection. *C. burnetii* was sonicated for 10 min before being added to each well at an MOI of 500. Coverslips were fixed with cold 100% methanol on ice for 5 min. Coverslips were then rinsed three times each with phosphate-buffered saline (PBS) and blocked for 1 h in 2% bovine serum albumin (BSA)–PBS. Next, coverslips were incubated with the appropriate primary antibodies diluted in block solution for 1 h and then rinsed in PBS three times and incubated with appropriate secondary antibodies diluted in block solution for 1 h. Coverslips were then rinsed three times in PBS and mounted onto glass slides using ProLong Gold antifade reagent (Life Technologies).

Primary antibodies were as follows: 1:10,000 rabbit anti-*C. burnetii* (4), 1:2,000 mouse anti-LAMP (H4A3-c; Developmental Studies Hybridoma Bank, University of Iowa), 1:200 mouse anti-LC3 (2G6; NanoTools), 1:200 CD63 mouse anti-CD63 (Developmental Studies Hybridoma Bank, University of Iowa), 1:200 rabbit anti-NDP52/Calcoco2 (Abcam), and 1:500 rabbit anti-p62/SQSTM1 (Abcam). Secondary antibodies were used at 1:2,000 and were Alexa Fluor goat anti-mouse 488, Alexa Fluor goat anti-rabbit 568, and Alexa Fluor goat anti-mouse 647 (all from Life Technologies). DAPI (4′,6-diamidino-2-phenylindole) (Life Technologies) was added with secondary antibodies at a dilution of 1:25,000. Coverslips were imaged on a Nikon Eclipse TE2000-S inverted fluorescence microscope with a 100×/1.4 numerical aperture objective lens. The microscope camera was a Photometrics CoolSNAP EZ camera controlled by SlideBook (version 6.0) software.

### YFP-LC3 HeLa infections and live-cell photos.

For experiments utilizing stable YFP-LC3 HeLa cells, 2 × 10^4^ cells were plated in 35-mm-diameter glass bottom dishes (MatTek) 2 days prior to infection in 5% FBS–DMEM without antibiotics. Cells were infected for 5 days before images of live infected cells were acquired using a 100×/1.4 numerical aperture objective lens on a Nikon Eclipse fluorescence microscope (described above). Bafilomycin A1 (Life Technologies) (100 ng/ml) was added to the media for 60 min prior to imaging.

### EM.

Stably expressing GFP-RFP-LC3 HeLa cells were infected with either wild-type or *cig2*::Tn *C. burnetii* for 5 days. Cells were fixed in 4% paraformaldehyde (PFA)–0.1% glutaraldehyde–PBS for 30 min followed by further fixation in 4% PFA for 1 h. They were rinsed in PBS, scraped, and resuspended in 10% gelatin. Chilled blocks were trimmed and placed in 2.3 M sucrose overnight on a rotor at 4°C. Samples were then transferred to aluminum pins and frozen rapidly in liquid nitrogen. The frozen blocks were cut on a Leica Cryo-EM UC6 UltraCut ultramicrotome. Sections (65 nm thick) were collected using the Tokuyasu method (50) and placed on carbon/Formvar-coated grids and floated in a dish of PBS for immunolabeling. Grids were placed section side down on drops of 0.1 M ammonium chloride to quench untreated aldehyde groups and then blocked for nonspecific binding on 1% fish skin gelatin–PBS. Single labeled grids were incubated with mouse anti-GFP antibody (Roche; 11814460001) using a 1:25 dilution, which required a rabbit anti-mouse bridge (Jackson Immuno Research Labs). Protein A gold (Utrecht Medical Center) (10-nm-diameter particles) was used as a secondary antibody. All grids were rinsed in PBS, fixed using 1% glutaraldehyde for 5 min, rinsed again, and transferred to a uranyl acetate (UA)-methylcellulose drop before being collected and dried. Grids were viewed on an FEI Tencai Biotwin transmission electron microscope (TEM) at 80 kV using a Morada charge-coupled device (CCD) camera and iTEM (Olympus) software. Quantification of gold beads was performed in ImageJ.

### *C. burnetii* growth curves.

Growth curve analyses were performed as described in Newton et al. (29). Briefly, 5 × 10^4^ HeLa cells were plated the day before infection. Cells were infected at an MOI of 50 for 4 h and then washed with PBS to remove excess bacteria. Samples were lysed in autoclaved distilled water prior to collection at each time point. Samples were processed for genomic DNA using an Illustra genomic bacterial prep mini spin kit (GE). Genome equivalents were quantified by qPCR.

### siRNA silencing experiments.

siRNA silencing was performed as previously described (51). siRNAs were ON-TARGETplus pools obtained from Dharmacon, except those used against NDP52, which were custom ordered from Sigma using the exact sequences described by Thurston et al. (19). HeLa cells were reverse transfected in a 24-well dish using 200 nM siRNA and Dharmafect transfection reagent and then lifted 2 days later and replated on coverslips. Cells were then infected with *C. burnetii* bacteria at an MOI of 500 for 3 days prior to fixing and staining performed as described above. LC3 immunoblotting was performed as described by Newton et al. (29), using anti-rabbit LC3 antibody (novus). For autophagy receptor immunoblotting, uninfected cells were lysed in 5× Laemmli buffer and loaded onto a 12% SDS-acrylamide gel. Samples were transferred to a polyvinylidene difluoride (PVDF) membrane at 20 V overnight at 4C. Membranes were blocked in 5% milk–PBS–0.05% Tween 20 for 1 h. Primary antibodies were 1:200 anti-rabbit NDP52 (Abcam), 1:500 anti-mouse p62 (Millipore), and anti-rabbit actin (Abcam). Secondary antibodies were used at 1:2,000 in a block and were either horseradish peroxidase (HRP) goat anti-mouse or HRP goat anti-rabbit (each from Invitrogen). For the TAX1BP1 immunoblot analysis, the membrane was blocked in 5% BSA–PBS–0.05% Tween 20 for 1 h and then incubated with anti-rabbit TAX1BP1 (1:1,000) in the same BSA blocking solution overnight at 4C. Membrane images were acquired using an ImageQuant LAS 4000 imager (GE).

### Generation of EGFP-expressing *C. burnetii* strains.

A pJB kanamycin plasmid (pJB_Kan) was cloned to express EGFP under the control of the *cbu0311* promoter. EGFP was generated by PCR from pEGFP-N2 (Clontech), and the *cbu0311* promoter was generated by PCR from NMII genomic DNA. The primers used to generate the *cbu0311* promoter were ATCGATTACAAGGATGACGATGACAAGGTCGACGATTATTAATTCAAACGGGTCAGGATT and ATGTCAAATCTCCGTTTTCAACTAAAGT. The primers used to generate the EGFP gene were GATTTTGTTTTAACTTTAGTTGAAAACGGAGATTTGACATGGTGAGCAAGGGCGAGG and GACGAGTTCTTCTGAAAGCTTGCATGCCTCAGTCGACTTACTTGTACAGCTCGTCCATG. Sequence- and ligation-independent cloning (SLIC) was performed to fuse the *cbu0311* promoter with the EGFP gene and to insert both pieces into the SalI site of pJB_Kan (52). The plasmid was then transformed into *C. burnetii* strains as previously described (29).

### Fusion assay coinfections.

After quantification of genome equivalents by qPCR, equal numbers of isogenic pairs of strains were mixed together to make stock at an MOI of 1,000. This stock was then diluted in 5% FBS–DMEM so that equivalent volumes (50 µl) of each mix could be added to the cells for infection for lesser MOIs. The resultant MOI represents the total number of bacteria added to cells for the two strains combined. For example, for an MOI of 1,000, MOIs of 500 for each strain in the pair were mixed together. Mixes of isogenic strains were sonicated for 10 min in a water bath sonicator prior to infection. HeLa cells were plated on coverslips in 24-well dishes as described above. Coverslips were rinsed with PBS before fixation was performed with 4% paraformaldehyde for 15 min on day 5 after infection. Coverslips were blocked in 2% BSA–0.5% saponin–PBS for 1 h. Coverslips were then stained with primary antibodies (using the anti-LAMP and anti-*Coxiella* antibodies as described above) under block conditions for 1 h. Coverslips were rinsed three times with PBS and then incubated with secondary antibodies under block conditions. Secondary antibodies were Alexa Fluor goat anti-mouse 647 and Alexa Fluor goat anti-rabbit 568 (both from Life Technologies). Coverslips were rinsed with PBS and mounted onto glass slides using ProLong Gold antifade reagent (Life Technologies). Coverslips were imaged on the Nikon microscope as described above. Infected cells were each quantified in two ways: for multiple vacuoles and for a fusion defect. A cell with a CCV fusion defect was defined as having multiple vacuoles that contained both GFP- and non-GFP-expressing bacteria in CCVs, with at least one vacuole that had only non-GFP-expressing bacteria. Three technical replicates were performed in each experiment. A total of 100 infected cells from each of the technical replicates were counted.

For coinfections with wild-type *C. burnetii* expressing EGFP and *cig2*::Tn (expressing mCherry from the transposon), strains were mixed at the appropriate MOIs and used to infect cells as described for the isogenic strain pairs. Coverslips were processed as described for the isogenic strain coinfections except that they were stained only for LAMP-1 and not with anti-*Coxiella* antibody. Infected cells were quantified as described above.

### Generation of Tn*7* complementation strains.

A single copy of *cig2* flanked by 250 bp of upstream genomic sequence (including the endogenous promoter) and 100 bp of downstream genomic sequence was cloned into the PstI site of pMiniTn*7*T-KAN (a gift from P. Beare and R. Heinzen at Rocky Mountain Laboratory) using SLIC. This plasmid was adapted from that previously described to contain a kanamycin resistance cassette in place of the chloramphenicol resistance cassette present in prior versions. A synthetic terminator (L3S2P21) was added to the plasmid using InFusion cloning by cutting with KpnI and using the primers CTTGGGCCCGGTACCAAATTCCAGAAAAGAGGCCTCCCGAAAGGGGGGCCTTTTTTCGTTTTGGTCCACTAGTCTCGCGAAGGCCTTG and CAAGGCCTTCGCGAGACTAGTGGACCAAAACGAAAAAAGGCCCCCCTTTCGGGAGGCCTCTTTTCTGGAATTTGGTACCGGGCCCAAG. The sequences of the primers used to add *cig2* to the plasmid were GCATGAGCTCACTAGTGGATCCCCCGGGCTGCAGATGTAATTTGAAGTGGAACTTGCATG and GAATTTGGTACCGGGCCCAAGCTTCTCGAGGAATTCCTGCAGGCGGGAATGACGGAACTG. The resultant plasmid was transformed into the wild-type and *cig2*::Tn *C. burnetii* strains with a helper plasmid (pTNS2-p1169-TnsA) as described by Beare et al. (35). The identities of the resultant strains were confirmed by sequencing a PCR product generated by amplifying the region next to the Tn*7* insertion site in *cig2*.

### *G. mellonella* infections and sample processing.

*Galleria mellonella* infections were performed as previously described (34), with the following changes: larvae were obtained from Vanderhorst Wholesale, Inc., separated into groups of 15 worms weighing between 0.2 and 0.3 g each, and injected using a sterile Hamilton syringe with 10^6^ genome equivalents for survival experiments. For survival assessment, culture tubes were processed in a blind manner before injection was performed in each of three independent experiments. For determinations of sublethal replication curves, three randomly selected larvae were collected every 48 h and stored at −20°C until processing. Total genomic DNA was purified from larvae using a Qiagen DNEasy blood and tissue kit with overnight lysis in buffer ATL plus proteinase K at 56°C, with larval lysates split between two purification columns and combined before quantification. *C. burnetii* genome equivalents were enumerated using qPCR to amplify the *dotA* gene. For hemocyte imaging, larvae were injected with 10^7^ genome equivalents of *C. burnetii* strains expressing GFP from plasmid pJB-311-GFP. On day 5 postinfection, hemolymph was collected as previously described (53). Hemocytes were fixed in 4% paraformaldehyde–PBS, permeabilized with 0.5% Triton X-100, and stained with Hoechst stain and Alexa Fluor 568-phalloidin (Thermo Fisher). Images were collected on a Nikon Eclipse TE2000-S inverted fluorescence microscope as described above at ×60 magnification, and 4-µm Z-projections were deconvolved and processed using SlideBook 6.0.

## SUPPLEMENTAL MATERIAL

Figure S1  CCVs formed by *cig2*::Tn display CD63. HeLa cells were infected with the indicated *C. burnetii* strains for 5 days and then fixed and stained with antibody to endogenous CD63. Download Figure S1, TIF file, 2.5 MB

Figure S2  GFP-expressing *C. burnetii* strains display green fluorescence during growth in CCVs. HeLa cells were infected for 5 days with the indicated *C. burnetii* strains. Cells were fixed and stained with DAPI and antibodies to *C. burnetii* and LAMP-1. Download Figure S2, TIF file, 2.4 MB

Figure S3  Autophagy is not required for *C. burnetii* growth in HeLa cells. The growth curve of indicated *C. burnetii* strains in HeLa cells is based on genomic equivalents quantified by qPCR. Download Figure S3, TIF file, 0.9 MB
