# Erratum for Kohler et al., Effector Protein Cig2 Decreases Host Tolerance of Infection by Directing Constitutive Fusion of Autophagosomes with the *Coxiella*-Containing Vacuole

**DOI:** 10.1128/mBio.01327-16

**Published:** 2016-09-06

**Authors:** Lara J. Kohler, Shawna C. O. Reed, Shireen A. Sarraf, David D. Arteaga, Hayley J. Newton, Craig R. Roy

**Affiliations:** a Department of Microbial Pathogenesis, Boyer Center for Molecular Medicine, Yale University School of Medicine, New Haven, Connecticut, USA; b Biochemistry Section, Surgical Neurology Branch, National Institute of Neurological Disorders and Stroke, National Institutes of Health, Bethesda, Maryland, USA; c Department of Microbiology and Immunology, University of Melbourne, at the Peter Doherty Institute for Infection and Immunity, Melbourne, Victoria, Australia

## ERRATUM

Volume 7, no. 4, doi:10.1128/mBio.01127-16, 2016. The second author’s name was spelled incorrectly in the article byline. The byline should appear as shown above.

