# Supplementary figures and images for: Effector Protein Cig2 Decreases Host Tolerance of Infection by Directing Constitutive Fusion of Autophagosomes with the Coxiella-Containing Vacuole
Source: mBio. 2016 Jul 19;7(4):e01127-16. doi: 10.1128/mBio.01127-16 (PMC4958265; doi:10.1128/mBio.01127-16)

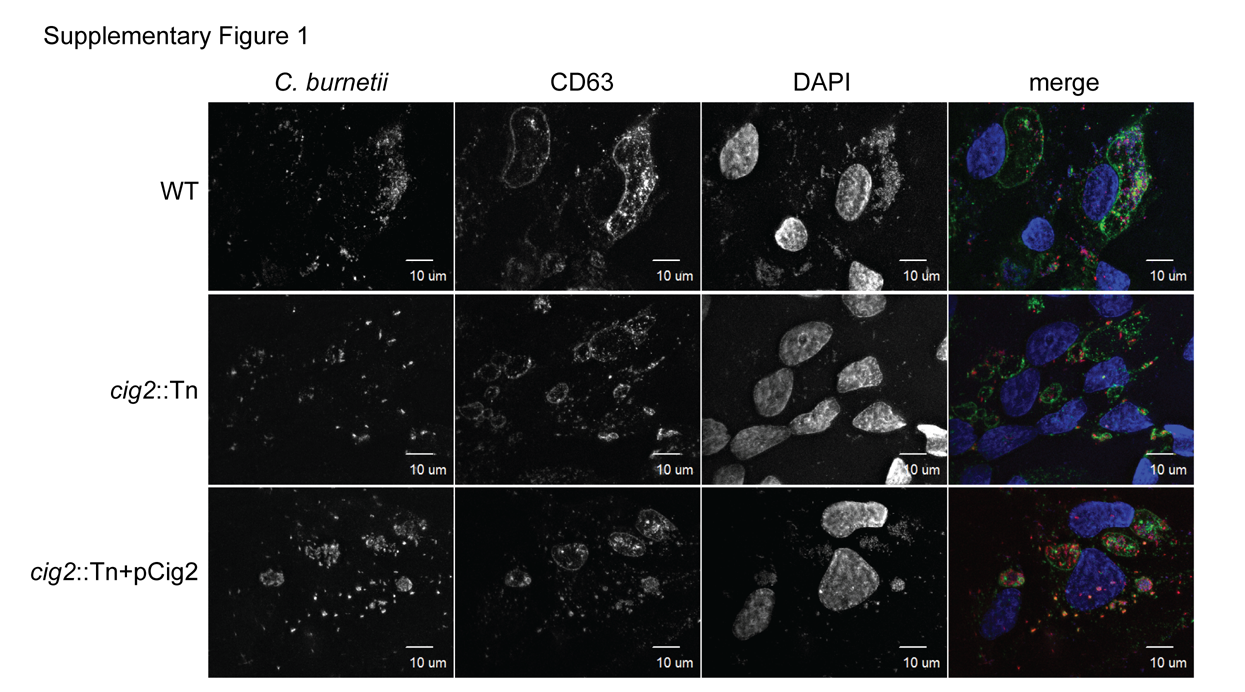

Supplement: Figure S1 — CCVs formed by cig2::Tn display CD63. HeLa cells were infected with the indicated C. burnetii strains for 5 days and then fixed and stained with antibody to endogenous CD63. Download [file mbo004162918sf1.tif]

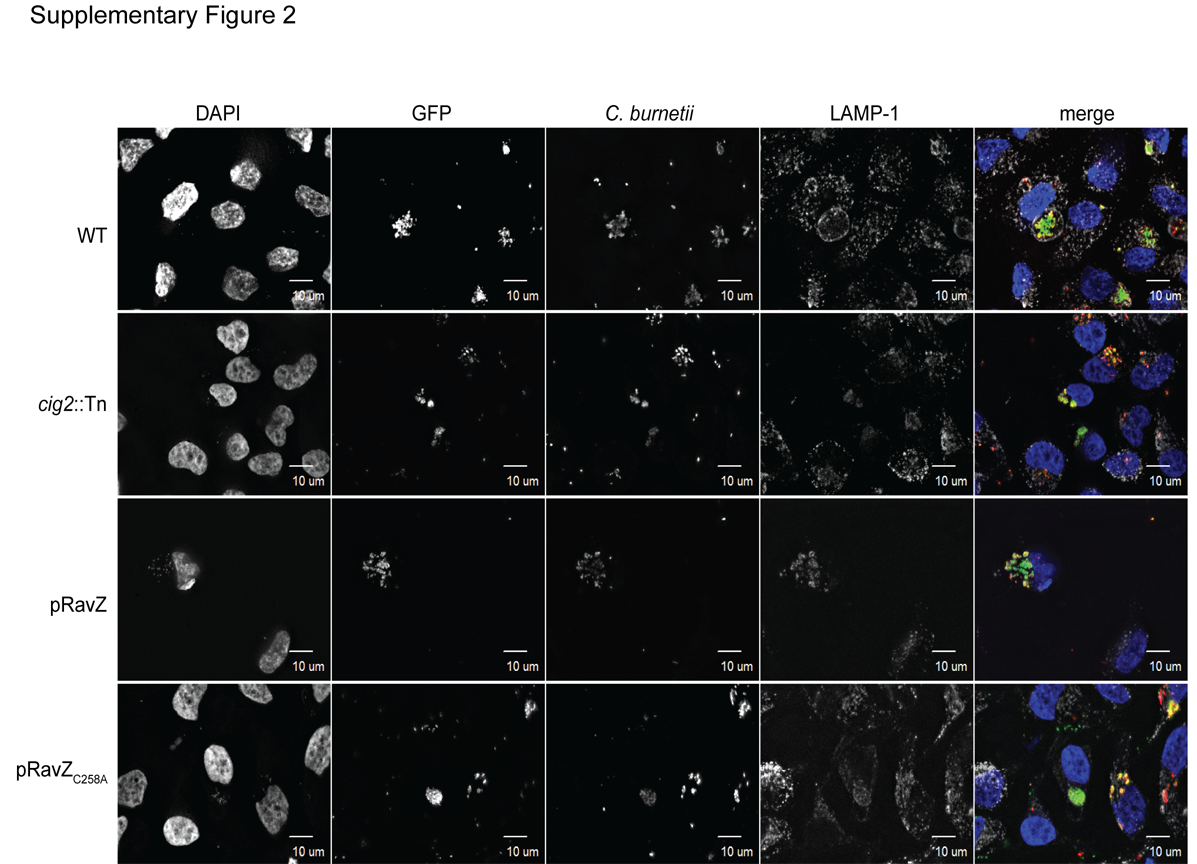

Supplement: Figure S2 — GFP-expressing C. burnetii strains display green fluorescence during growth in CCVs. HeLa cells were infected for 5 days with the indicated C. burnetii strains. Cells were fixed and stained with DAPI and antibodies to C. burnetii and LAMP-1. Download [file mbo004162918sf2.tif]

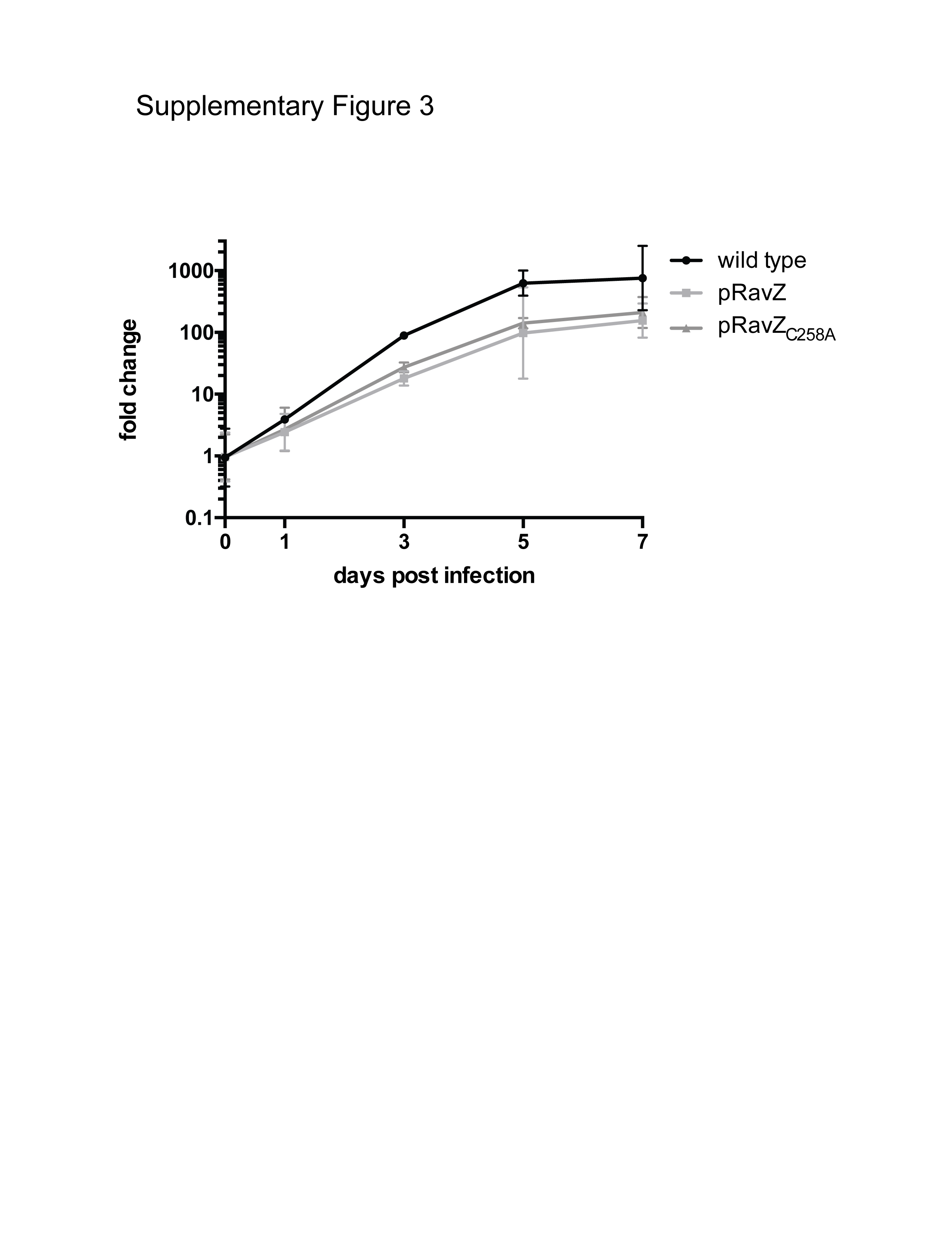

Supplement: Figure S3 — Autophagy is not required for C. burnetii growth in HeLa cells. The growth curve of indicated C. burnetii strains in HeLa cells is based on genomic equivalents quantified by qPCR. Download [file mbo004162918sf3.tif]
